# Irradiated fuel salt data library for a molten salt reactor produced with Serpent2 and SOURCES 4C codes

**DOI:** 10.1016/j.dib.2023.109817

**Published:** 2023-11-17

**Authors:** Vaibhav Mishra, Zsolt Elter, Erik Branger, Sophie Grape, Sorouche Mirmiran

**Affiliations:** a Department of Physics and Astronomy, Applied Nuclear Physics Division, Uppsala University, Uppsala, Sweden; b Seaborg Technologies, Titangade 11 2200, Copenhagen, Denmark

**Keywords:** Nuclear safeguards, Safeguards verification, Molten salt reactors, Spent fuel, Burnup, Serpent, SOURCES 4C

## Abstract

This paper describes the creation and description of a nuclear fuel isotopics dataset for irradiated fuel salt from a Molten Salt Reactor (MSR). The dataset has been created using simulations carried out using the Monte-Carlo particle transport code, Serpent 2.1.32 (released February 24, 2021) and the calculation code SOURCES 4C (released October 09, 2002) for computing properties of irradiated molten fuel salt. The dataset comprises isotopic mass densities of 1362 isotopes (including fission products and major and minor actinides) and their corresponding contributions to decay heat, gamma activity, and spontaneous fission rates computed by Serpent 2.1.32 as well as overall neutron emission rates from spontaneous fission and (ɑ, n) reactions computed by SOURCES 4C. These quantities are computed for a model MSR core utilizing a full-core 3D model of the Seaborg Compact Molten Salt Reactor (CMSR). The dataset spans a wide range of values of burnup (BU), initial enrichment (IE) and cooling time (CT) over which the above-mentioned quantities are reported.

The structure of the dataset includes isotopic mass densities (in g/cm^3^), followed by isotope-wise contributions to decay heat (denoted by suffix *‘_DH’* and reported in Watts), gamma photon emission rates (denoted by suffix *‘_GS’* and reported photons per second), and spontaneous fission rates (denoted by suffix *‘_SF’* and reported in fissions per second). In addition to these columns, the data also includes total neutron emission rates from 1) spontaneous fission (denoted by *‘SF’* and reported in neutrons per second per cm^3^), and 2) (ɑ, n) reactions (denoted by *‘AN’* and reported in neutrons per second per cm^3^). In total, the dataset has 310,575 rows of different combinations of fuel burnup, initial enrichment, and cooling time (BIC) values spanning the realistic possible range of these parameters. The dataset is made available for public use in a comma-separated value file that can be easily read using one of the numerous popular data analysis tools such as NumPy or Pandas.

Specifications Table

**Table:** 

Subject	Nuclear Energy and Engineering
Specific subject area	Molten Salt Reactors, nuclear fuel modeling, burnup studies, irradiated fuel salt, Seaborg CMSR, nuclear safeguards.
Data format	Raw and filtered
Type of data	Table
Data collection	The dataset has been compiled using information extracted from output files (dep.m) generated by the Serpent calculations of fuel salt irradiation in an MSR core and `*tape 6*’ files generated by SOURCES 4C.
Data source location	Uppsala University, Uppsala, Sweden.
Data availability	Uploaded to Mendeley Data at https://data.mendeley.com/datasets/fbnmwnyg92/1 DOI is https://doi.org/10.17632/fbnmwnyg92.1

## 1 Value of the Data

• The irradiated fuel salt database was created with the purpose of documenting properties such as isotopic mass densities, gamma activity, decay heat and spontaneous fission rates and associated neutron emissions from both, spontaneous fissions as well as the (ɑ, n) reactions in the spent fuel salt over a reasonably wide range of BIC parameters.
• The primary motivation for this work stems from the lack of availability of such fuel databases for MSRs. Such libraries exist for traditional Light Water Reactor (LWR) fuel (like [1] and [2]); however corresponding libraries are scarce for MSRs.
• The data has been produced using codes Serpent 2.1.32 [3] and SOURCES 4C [4]. Previously developed LWR fuel libraries have also been developed using simulations of fuel irradiation with similar Monte-Carlo particle transport codes i.e. Serpent and SCALE [5].
• This database extends a methodology similar to the one used in [2] to create a library of nuclear safeguards-centric fuel signatures which could be used in MSR-related research such as accounting and verifying nuclear material in MSRs in the future.
• At the present time, the existence of regulatory framework and guidelines of how and when to verify irradiated fuel salts is scarce and the experience of the industry in dealing with highly radioactive and corrosive fuel salts after discharge is severely limited. Therefore, a fuel database with signatures that could be considered for verifying spent fuel of such unique nature could be valuable.
• Since LWR fuel libraries cannot be directly used for MSR fuel owing to differences in composition of fuel, reactor physics, and operation, fuel libraries such as these will prove to be useful for safeguards-relevant analyses for irradiated salts.
• The dataset described in this work is based on a molten salt reactor design [6] proposed by Seaborg Technologies (headquartered in Copenhagen, Denmark) and the technical specifications of the reactor are discussed in the following sections.

## 2 Background

As more advanced Generation-IV reactors such as MSRs enter commercial deployment in the coming decades, it is expected that regulatory authorities are likely to face nuclear safeguards-related challenges associated with spent fuel from these reactors. Much about the nature of spent fuel from these reactors remains to be studied and is currently an active area of research. More recently, novel techniques such as machine learning have been applied to verification of spent fuel from commercial LWRs. Since these techniques rely on large amounts of data, there has been a dire need for the development of datasets that could be used for such purposes. The relative lack of such datasets for MSRs is a problem that has been addressed and underscored in this paper.

The dataset presented in this paper, on its own, is expected to provide practical information on the nature of irradiated fuel salt after discharge from the core. Information such as the mass densities of fission products (and their decay products) as well as those of minor and major actinides, their contributions to decay heat, gamma activity, spontaneous fission rates, and neutron emissions at various combinations of salt BIC are available for direct use from the dataset.

In the future, it is expected that the dataset will be used to train machine learning algorithms on these signatures as input features to predict fuel salt's BIC parameters, fissile mass etc.

## 3 Data Description

The dataset is supplied as a comma-separated value (csv) file consisting of 1362 columns, each corresponding to the mass density of a specific isotope. Similarly, there are 1362 columns of each of the fuel salt signatures such as the decay heat, gamma source rate and spontaneous fission rate. The columns describing decay heat, spontaneous fission rates and gamma source rates contributions use the following naming convention: *‘Isotope_XX’* where ‘Isotope’ refers to the radionuclide in question written in ‘name’ notation [3] and the suffix *‘_XX’* could imply decay heat contributions (written as *‘_DH’*), or gamma source contributions (written as *‘_GS’*) or spontaneous fission contributions (written as *‘_SF’*). These quantities are reported at 75 BU, 41 IE, and 101 CT steps. Therefore, in total, there are 5450 columns of data for each BIC combination with 310,575 possible combinations of BIC. The different parameters included in the dataset are further described in Table 1.

**Table 1:** Description of various columns corresponding to quantities included in the dataset.

Parameter column	Parameter description
‘BU’	Burnup (in MWd/kgU)
‘IE’	Initial enrichment (in wt. % U-235)
‘CT’	Cooling time (in days)
Isotopes	1362 columns corresponding to all mass densities of isotopes tracked by Serpent (in g/cm^3^)
‘Isotope_DH’	Isotope specific decay heat (in Watts) included in 1362 columns
‘Isotope_GS’	Isotope specific gamma emission rate (photons/second) included in 1362 columns
‘Isotope_SF’	Isotope specific spontaneous fission rate (fissions/second) included in 1362 columns
‘SF’	Total neutron emissions from all spontaneous fission reactions occurring in the irradiated salt
‘AN’	Total neutron emissions from (ɑ, n) reactions occurring in the irradiated salt

The full list of nuclides (sorted and grouped alphabetically) included in the dataset is given below:

A:

Ac-225’, ‘Ac-226’, ‘Ac-227’, ‘Ag-108’, ‘Ag-108m’, ‘Ag-109’, ‘Ag-109m’, ‘Ag-110’, ‘Ag-110m’, ‘Ag-111’, ‘Ag-111m’, ‘Ag-112’, ‘Ag-113’, ‘Ag-113m’, ‘Ag-114’, ‘Ag-114m’, ‘Ag-115’, ‘Ag-115m’, ‘Ag-116’, ‘Ag-116m’, ‘Ag-117’, ‘Ag-117m’, ‘Ag-118’, ‘Ag-118m’, ‘Ag-119’, ‘Ag-119m’, ‘Ag-120’, ‘Ag-120m’, ‘Ag-121’, ‘Ag-122’, ‘Ag-122m’, ‘Ag-123’, ‘Ag-124’, ‘Ag-124m’, ‘Ag-125’, ‘Ag-126’, ‘Ag-127’, ‘Ag-128’, ‘Ag-129’, ‘Ag-130’, ‘Am-241’, ‘Am-242’, ‘Am-242m’, ‘Am-243’, ‘Am-244’, ‘Am-244m’, ‘As-73’, ‘As-74’, ‘As-75’, ‘As-75m’, ‘As-76’, ‘As-77’, ‘As-78’, ‘As-79’, ‘As-80’, ‘As-81’, ‘As-82’, ‘As-82m’, ‘As-83’, ‘As-84’, ‘As-84m’, ‘As-85’, ‘As-86’, ‘As-87’, ‘As-88’, ‘As-89’, ‘As-90’, ‘As-91’, ‘Au-195’, ‘Au-196’, ‘Au-196m’, ‘Au-197’, ‘Au-197m’, ‘Au-198’, ‘Au-198m’, ‘Au-199’, ‘Au-200’, ‘Au-200m’,

B:

‘B-10’, ‘B-12’, ‘B-9’, ‘Ba-133’, ‘Ba-133m’, ‘Ba-134’, ‘Ba-135’, ‘Ba-135m’, ‘Ba-136’, ‘Ba-136m’, ‘Ba-137’, ‘Ba-137m’, ‘Ba-138’, ‘Ba-139’, ‘Ba-140’, ‘Ba-141’, ‘Ba-142’, ‘Ba-143’, ‘Ba-144’, ‘Ba-145’, ‘Ba-146’, ‘Ba-147’, ‘Ba-148’, ‘Ba-149’, ‘Ba-150’, ‘Ba-151’, ‘Ba-152’, ‘Ba-153’, ‘Be-10’, ‘Be-11’, ‘Be-12’, ‘Be-8’, ‘Be-9’, ‘Bk-249’, ‘Br-78’, ‘Br-79’, ‘Br-79m’, ‘Br-80’, ‘Br-80m’, ‘Br-81’, ‘Br-82’, ‘Br-82m’, ‘Br-83’, ‘Br-84’, ‘Br-84m’, ‘Br-85’, ‘Br-86’, ‘Br-87’, ‘Br-88’, ‘Br-89’, ‘Br-90’, ‘Br-91’, ‘Br-92’, ‘Br-93’, ‘Br-94’, ‘Br-95’, ‘Br-96’,

C:

‘C-14’, ‘C-15’, ‘C-8’, ‘Ca-50’, ‘Ca-51’, ‘Ca-52’, ‘Ca-53’, ‘Ca-54’, ‘Cd-111’, ‘Cd-111m’, ‘Cd-112’, ‘Cd-113’, ‘Cd-113m’, ‘Cd-114’, ‘Cd-115’, ‘Cd-115m’, ‘Cd-116’, ‘Cd-117’, ‘Cd-117m’, ‘Cd-118’, ‘Cd-119’, ‘Cd-119m’, ‘Cd-120’, ‘Cd-121’, ‘Cd-121m’, ‘Cd-122’, ‘Cd-123’, ‘Cd-123m’, ‘Cd-124’, ‘Cd-125’, ‘Cd-125m’, ‘Cd-126’, ‘Cd-127’, ‘Cd-128’, ‘Cd-129’, ‘Cd-129m’, ‘Cd-130’, ‘Cd-131’, ‘Cd-132’, ‘Ce-138’, ‘Ce-138m’, ‘Ce-139’, ‘Ce-139m’, ‘Ce-140’, ‘Ce-141’, ‘Ce-142’, ‘Ce-143’, ‘Ce-144’, ‘Ce-145’, ‘Ce-146’, ‘Ce-147’, ‘Ce-148’, ‘Ce-149’, ‘Ce-150’, ‘Ce-151’, ‘Ce-152’, ‘Ce-153’, ‘Ce-154’, ‘Ce-155’, ‘Ce-156’, ‘Ce-157’, ‘Cf-249’, ‘Cf-251’, ‘Cm-240’, ‘Cm-241’, ‘Cm-242’, ‘Cm-243’, ‘Cm-244’, ‘Cm-245’, ‘Cm-246’, ‘Cm-247’, ‘Cm-248’, ‘Cm-249’, ‘Cm-250’, ‘Co-59’, ‘Co-60’, ‘Co-60m’, ‘Co-61’, ‘Co-62’, ‘Co-62m’, ‘Co-63’, ‘Co-64’, ‘Co-65’, ‘Co-66’, ‘Co-67’, ‘Co-68’, ‘Co-68m’, ‘Co-69’, ‘Co-70’, ‘Co-70m’, ‘Co-71’, ‘Co-72’, ‘Co-73’, ‘Co-74’, ‘Co-75’, ‘Cr-52’, ‘Cr-53’, ‘Cr-54’, ‘Cr-55’, ‘Cr-56’, ‘Cr-57’, ‘Cr-58’, ‘Cr-59’, ‘Cr-60’, ‘Cr-61’, ‘Cr-62’, ‘Cr-63’, ‘Cr-64’, ‘Cr-65’, ‘Cr-66’, ‘Cr-67’, ‘Cs-131’, ‘Cs-132’, ‘Cs-133’, ‘Cs-134’, ‘Cs-134m’, ‘Cs-135’, ‘Cs-135m’, ‘Cs-136’, ‘Cs-136m’, ‘Cs-137’, ‘Cs-138’, ‘Cs-138m’, ‘Cs-139’, ‘Cs-140’, ‘Cs-141’, ‘Cs-142’, ‘Cs-143’, ‘Cs-144’, ‘Cs-144m’, ‘Cs-145’, ‘Cs-146’, ‘Cs-147’, ‘Cs-148’, ‘Cs-149’, ‘Cs-150’, ‘Cu-64’, ‘Cu-65’, ‘Cu-66’, ‘Cu-67’, ‘Cu-68’, ‘Cu-68m’, ‘Cu-69’, ‘Cu-70’, ‘Cu-70m’, ‘Cu-71’, ‘Cu-72’, ‘Cu-73’, ‘Cu-74’, ‘Cu-75’, ‘Cu-76’, ‘Cu-76m’, ‘Cu-77’, ‘Cu-78’, ‘Cu-79’, ‘Cu-80’,

D:

‘Dy-159’, ‘Dy-160’, ‘Dy-161’, ‘Dy-162’, ‘Dy-163’, ‘Dy-164’, ‘Dy-165’, ‘Dy-165m’, ‘Dy-166’, ‘Dy-167’, ‘Dy-168’, ‘Dy-169’, ‘Dy-170’, ‘Dy-171’, ‘Dy-172’, ‘Dy-173’,

E:

‘Er-164’, ‘Er-165’, ‘Er-166’, ‘Er-167’, ‘Er-167m’, ‘Er-168’, ‘Er-169’, ‘Er-170’, ‘Er-171’, ‘Er-172’, ‘Er-173’, ‘Er-174’, ‘Er-175’, ‘Er-176’, ‘Er-177’, ‘Eu-151’, ‘Eu-152’, ‘Eu-152m’, ‘Eu-153’, ‘Eu-154’, ‘Eu-154m’, ‘Eu-155’, ‘Eu-156’, ‘Eu-157’, ‘Eu-158’, ‘Eu-159’, ‘Eu-160’, ‘Eu-161’, ‘Eu-162’, ‘Eu-163’, ‘Eu-164’, ‘Eu-165’, ‘Eu-166’, ‘Eu-167’,

F:

‘Fe-56’, ‘Fe-57’, ‘Fe-58’, ‘Fe-59’, ‘Fe-60’, ‘Fe-61’, ‘Fe-62’, ‘Fe-63’, ‘Fe-64’, ‘Fe-65’, ‘Fe-66’, ‘Fe-67’, ‘Fe-68’, ‘Fe-69’, ‘Fe-70’, ‘Fe-71’, ‘Fe-72’,

G:

‘Ga-68’, ‘Ga-69’, ‘Ga-70’, ‘Ga-71’, ‘Ga-72’, ‘Ga-72m’, ‘Ga-73’, ‘Ga-74’, ‘Ga-74m’, ‘Ga-75’, ‘Ga-76’, ‘Ga-77’, ‘Ga-78’, ‘Ga-79’, ‘Ga-80’, ‘Ga-81’, ‘Ga-82’, ‘Ga-83’, ‘Ga-84’, ‘Ga-85’, ‘Ga-86’, ‘Gd-152’, ‘Gd-154’, ‘Gd-155’, ‘Gd-155m’, ‘Gd-156’, ‘Gd-157’, ‘Gd-158’, ‘Gd-159’, ‘Gd-160’, ‘Gd-161’, ‘Gd-162’, ‘Gd-163’, ‘Gd-164’, ‘Gd-165’, ‘Gd-166’, ‘Gd-167’, ‘Gd-168’, ‘Gd-169’, ‘Ge-70’, ‘Ge-71’, ‘Ge-71m’, ‘Ge-72’, ‘Ge-73’, ‘Ge-73m’, ‘Ge-74’, ‘Ge-75’, ‘Ge-75m’, ‘Ge-76’, ‘Ge-77’, ‘Ge-77m’, ‘Ge-78’, ‘Ge-79’, ‘Ge-79m’, ‘Ge-80’, ‘Ge-81’, ‘Ge-81m’, ‘Ge-82’, ‘Ge-83’, ‘Ge-84’, ‘Ge-85’, ‘Ge-86’, ‘Ge-87’, ‘Ge-88’, ‘Ge-89’,

H:

‘H-1’, ‘H-2’, ‘H-3’, ‘He-4’, ‘He-6’, ‘He-8’, ‘Hf-175’, ‘Hf-176’, ‘Hf-177’, ‘Hf-177m’, ‘Hf-178’, ‘Hf-178m’, ‘Hf-179’, ‘Hf-179m’, ‘Hf-180’, ‘Hf-180m’, ‘Hf-181’, ‘Hf-182’, ‘Hf-182m’, ‘Hf-183’, ‘Hf-184’, ‘Hf-184m’, ‘Hf-185’, ‘Hf-186’, ‘Hf-187’, ‘Hf-188’, ‘Hg-198’, ‘Hg-199’, ‘Hg-199m’, ‘Hg-200’, ‘Ho-161’, ‘Ho-161m’, ‘Ho-162’, ‘Ho-162m’, ‘Ho-163’, ‘Ho-163m’, ‘Ho-164’, ‘Ho-164m’, ‘Ho-165’, ‘Ho-166’, ‘Ho-166m’, ‘Ho-167’, ‘Ho-168’, ‘Ho-168m’, ‘Ho-169’, ‘Ho-170’, ‘Ho-170m’, ‘Ho-171’, ‘Ho-172’, ‘Ho-173’, ‘Ho-174’, ‘Ho-175’,

I:

‘I-126’, ‘I-127’, ‘I-128’, ‘I-129’, ‘I-130’, ‘I-130m’, ‘I-131’, ‘I-132’, ‘I-132m’, ‘I-133’, ‘I-133m’, ‘I-134’, ‘I-134m’, ‘I-135’, ‘I-136’, ‘I-136m’, ‘I-137’, ‘I-138’, ‘I-139’, ‘I-140’, ‘I-141’, ‘I-142’, ‘I-143’, ‘I-144’, ‘In-113’, ‘In-113m’, ‘In-114’, ‘In-114m’, ‘In-115’, ‘In-115m’, ‘In-116’, ‘In-116m’, ‘In-117’, ‘In-117m’, ‘In-118’, ‘In-118m’, ‘In-119’, ‘In-119m’, ‘In-120’, ‘In-120m’, ‘In-121’, ‘In-121m’, ‘In-122’, ‘In-122m’, ‘In-123’, ‘In-123m’, ‘In-124’, ‘In-124m’, ‘In-125’, ‘In-125m’, ‘In-126’, ‘In-126m’, ‘In-127’, ‘In-127m’, ‘In-128’, ‘In-128m’, ‘In-129’, ‘In-129m’, ‘In-130’, ‘In-130m’, ‘In-131’, ‘In-131m’, ‘In-132’, ‘In-133’, ‘In-133m’, ‘In-134’, ‘In-135’, ‘Ir-189’, ‘Ir-189m’, ‘Ir-190’, ‘Ir-190m’, ‘Ir-191’, ‘Ir-191m’, ‘Ir-192’, ‘Ir-192m’, ‘Ir-193’, ‘Ir-193m’, ‘Ir-194’, ‘Ir-194m’, ‘Ir-195’, ‘Ir-195m’, ‘Ir-196’, ‘Ir-196m’, ‘Ir-197’, ‘Ir-197m’,

K:

‘K-50’, ‘K-51’, ‘Kr-80’, ‘Kr-81’, ‘Kr-81m’, ‘Kr-82’, ‘Kr-83’, ‘Kr-83m’, ‘Kr-84’, ‘Kr-85’, ‘Kr-85m’, ‘Kr-86’, ‘Kr-87’, ‘Kr-88’, ‘Kr-89’, ‘Kr-90’, ‘Kr-91’, ‘Kr-92’, ‘Kr-93’, ‘Kr-94’, ‘Kr-95’, ‘Kr-96’, ‘Kr-97’, ‘Kr-98’, ‘Kr-99’,

L:

‘La-136’, ‘La-136m’, ‘La-137’, ‘La-138’, ‘La-139’, ‘La-140’, ‘La-141’, ‘La-142’, ‘La-143’, ‘La-144’, ‘La-145’, ‘La-146’, ‘La-146m’, ‘La-147’, ‘La-148’, ‘La-149’, ‘La-150’, ‘La-151’, ‘La-152’, ‘La-153’, ‘La-154’, ‘La-155’, ‘Li-7’, ‘Li-8’, ‘Li-9’, ‘Lu-172’, ‘Lu-173’, ‘Lu-174’, ‘Lu-174m’, ‘Lu-175’, ‘Lu-176’, ‘Lu-176m’, ‘Lu-177’, ‘Lu-177m’, ‘Lu-178’, ‘Lu-178m’, ‘Lu-179’, ‘Lu-179m’, ‘Lu-180’, ‘Lu-180m’, ‘Lu-181’, ‘Lu-182’, ‘Lu-183’, ‘Lu-184’,

M:

‘Mn-54’, ‘Mn-55’, ‘Mn-56’, ‘Mn-57’, ‘Mn-58’, ‘Mn-58m’, ‘Mn-59’, ‘Mn-60’, ‘Mn-60m’, ‘Mn-61’, ‘Mn-62’, ‘Mn-62m’, ‘Mn-63’, ‘Mn-64’, ‘Mn-65’, ‘Mn-66’, ‘Mn-67’, ‘Mn-68’, ‘Mn-69’, ‘Mo-100’, ‘Mo-101’, ‘Mo-102’, ‘Mo-103’, ‘Mo-104’, ‘Mo-105’, ‘Mo-106’, ‘Mo-107’, ‘Mo-108’, ‘Mo-109’, ‘Mo-110’, ‘Mo-111’, ‘Mo-112’, ‘Mo-113’, ‘Mo-114’, ‘Mo-115’, ‘Mo-95’, ‘Mo-96’, ‘Mo-97’, ‘Mo-98’, ‘Mo-99’,

N:

‘Nb-100’, ‘Nb-100m’, ‘Nb-101’, ‘Nb-102’, ‘Nb-102m’, ‘Nb-103’, ‘Nb-104’, ‘Nb-104m’, ‘Nb-105’, ‘Nb-106’, ‘Nb-107’, ‘Nb-108’, ‘Nb-109’, ‘Nb-110’, ‘Nb-111’, ‘Nb-112’, ‘Nb-93’, ‘Nb-93m’, ‘Nb-94’, ‘Nb-94m’, ‘Nb-95’, ‘Nb-95m’, ‘Nb-96’, ‘Nb-97’, ‘Nb-97m’, ‘Nb-98’, ‘Nb-98m’, ‘Nb-99’, ‘Nb-99m’, ‘Nd-142’, ‘Nd-143’, ‘Nd-144’, ‘Nd-145’, ‘Nd-146’, ‘Nd-147’, ‘Nd-148’, ‘Nd-149’, ‘Nd-150’, ‘Nd-151’, ‘Nd-152’, ‘Nd-153’, ‘Nd-154’, ‘Nd-155’, ‘Nd-156’, ‘Nd-157’, ‘Nd-158’, ‘Nd-159’, ‘Nd-160’, ‘Nd-161’, ‘Ne-21’, ‘Ni-61’, ‘Ni-62’, ‘Ni-63’, ‘Ni-64’, ‘Ni-65’, ‘Ni-66’, ‘Ni-67’, ‘Ni-68’, ‘Ni-69’, ‘Ni-69m’, ‘Ni-70’, ‘Ni-71’, ‘Ni-72’, ‘Ni-73’, ‘Ni-74’, ‘Ni-75’, ‘Ni-76’, ‘Ni-77’, ‘Ni-78’, ‘Np-235’, ‘Np-236’, ‘Np-237’, ‘Np-238’, ‘Np-239’,

O:

‘Os-186’, ‘Os-187’, ‘Os-188’, ‘Os-189’, ‘Os-189m’, ‘Os-190’, ‘Os-190m’, ‘Os-191’, ‘Os-191m’, ‘Os-192’, ‘Os-192m’, ‘Os-193’, ‘Os-194’, ‘Os-195’, ‘Os-196’,

P:

‘Pa-231’, ‘Pa-232’, ‘Pa-233’, ‘Pd-105’, ‘Pd-106’, ‘Pd-107’, ‘Pd-107m’, ‘Pd-108’, ‘Pd-109’, ‘Pd-109m’, ‘Pd-110’, ‘Pd-111’, ‘Pd-111m’, ‘Pd-112’, ‘Pd-113’, ‘Pd-113m’, ‘Pd-114’, ‘Pd-115’, ‘Pd-115m’, ‘Pd-116’, ‘Pd-117’, ‘Pd-117m’, ‘Pd-118’, ‘Pd-119’, ‘Pd-120’, ‘Pd-121’, ‘Pd-122’, ‘Pd-123’, ‘Pd-124’, ‘Pm-146’, ‘Pm-147’, ‘Pm-148’, ‘Pm-148m’, ‘Pm-149’, ‘Pm-150’, ‘Pm-151’, ‘Pm-152’, ‘Pm-152m’, ‘Pm-153’, ‘Pm-154’, ‘Pm-154m’, ‘Pm-155’, ‘Pm-156’, ‘Pm-157’, ‘Pm-158’, ‘Pm-159’, ‘Pm-160’, ‘Pm-161’, ‘Pm-162’, ‘Pm-163’, ‘Pr-141’, ‘Pr-142’, ‘Pr-142m’, ‘Pr-143’, ‘Pr-144’, ‘Pr-144m’, ‘Pr-145’, ‘Pr-146’, ‘Pr-147’, ‘Pr-148’, ‘Pr-148m’, ‘Pr-149’, ‘Pr-150’, ‘Pr-151’, ‘Pr-152’, ‘Pr-153’, ‘Pr-154’, ‘Pr-155’, ‘Pr-156’, ‘Pr-157’, ‘Pr-158’, ‘Pr-159’, ‘Pt-192’, ‘Pt-193’, ‘Pt-193m’, ‘Pt-194’, ‘Pt-195’, ‘Pt-195m’, ‘Pt-196’, ‘Pt-197’, ‘Pt-197m’, ‘Pt-198’, ‘Pt-199’, ‘Pt-199m’, ‘Pu-236’, ‘Pu-237’, ‘Pu-238’, ‘Pu-239’, ‘Pu-240’, ‘Pu-241’, ‘Pu-242’, ‘Pu-243’, ‘Pu-244’, ‘Pu-246’,

R:

‘Rb-100’, ‘Rb-101’, ‘Rb-83’, ‘Rb-83m’, ‘Rb-84’, ‘Rb-84m’, ‘Rb-85’, ‘Rb-86’, ‘Rb-86m’, ‘Rb-87’, ‘Rb-88’, ‘Rb-89’, ‘Rb-90’, ‘Rb-90m’, ‘Rb-91’, ‘Rb-92’, ‘Rb-93’, ‘Rb-94’, ‘Rb-95’, ‘Rb-96’, ‘Rb-96m’, ‘Rb-97’, ‘Rb-98’, ‘Rb-98m’, ‘Rb-99’, ‘Re-183’, ‘Re-184’, ‘Re-184m’, ‘Re-185’, ‘Re-186’, ‘Re-186m’, ‘Re-187’, ‘Re-188’, ‘Re-188m’, ‘Re-189’, ‘Re-190’, ‘Re-190m’, ‘Re-191’, ‘Re-192’, ‘Re-193’, ‘Re-194’, ‘Rh-103’, ‘Rh-103m’, ‘Rh-104’, ‘Rh-104m’, ‘Rh-105’, ‘Rh-105m’, ‘Rh-106’, ‘Rh-106m’, ‘Rh-107’, ‘Rh-108’, ‘Rh-108m’, ‘Rh-109’, ‘Rh-110’, ‘Rh-110m’, ‘Rh-111’, ‘Rh-112’, ‘Rh-112m’, ‘Rh-113’, ‘Rh-114’, ‘Rh-114m’, ‘Rh-115’, ‘Rh-116’, ‘Rh-116m’, ‘Rh-117’, ‘Rh-118’, ‘Rh-119’, ‘Rh-120’, ‘Rh-121’, ‘Rh-122’, ‘Ru-100’, ‘Ru-101’, ‘Ru-102’, ‘Ru-103’, ‘Ru-103m’, ‘Ru-104’, ‘Ru-105’, ‘Ru-106’, ‘Ru-107’, ‘Ru-108’, ‘Ru-109’, ‘Ru-110’, ‘Ru-111’, ‘Ru-112’, ‘Ru-113’, ‘Ru-113m’, ‘Ru-114’, ‘Ru-115’, ‘Ru-116’, ‘Ru-117’, ‘Ru-118’, ‘Ru-119’, ‘Ru-120’,

S:

‘Sb-118’, ‘Sb-118m’, ‘Sb-119’, ‘Sb-119m’, ‘Sb-120’, ‘Sb-120m’, ‘Sb-121’, ‘Sb-122’, ‘Sb-122m’, ‘Sb-123’, ‘Sb-124’, ‘Sb-124m’, ‘Sb-125’, ‘Sb-126’, ‘Sb-126m’, ‘Sb-127’, ‘Sb-128’, ‘Sb-128m’, ‘Sb-129’, ‘Sb-129m’, ‘Sb-130’, ‘Sb-130m’, ‘Sb-131’, ‘Sb-132’, ‘Sb-132m’, ‘Sb-133’, ‘Sb-134’, ‘Sb-134m’, ‘Sb-135’, ‘Sb-136’, ‘Sb-137’, ‘Sb-138’, ‘Sb-139’, ‘Sc-50’, ‘Sc-50m’, ‘Sc-51’, ‘Sc-52’, ‘Sc-53’, ‘Sc-54’, ‘Sc-55’, ‘Sc-56’, ‘Sc-57’, ‘Se-75’, ‘Se-76’, ‘Se-77’, ‘Se-77m’, ‘Se-78’, ‘Se-79’, ‘Se-79m’, ‘Se-80’, ‘Se-81’, ‘Se-81m’, ‘Se-82’, ‘Se-83’, ‘Se-83m’, ‘Se-84’, ‘Se-85’, ‘Se-86’, ‘Se-87’, ‘Se-88’, ‘Se-89’, ‘Se-90’, ‘Se-91’, ‘Se-92’, ‘Se-93’, ‘Se-94’, ‘Sm-147’, ‘Sm-148’, ‘Sm-149’, ‘Sm-150’, ‘Sm-151’, ‘Sm-152’, ‘Sm-153’, ‘Sm-153m’, ‘Sm-154’, ‘Sm-155’, ‘Sm-156’, ‘Sm-157’, ‘Sm-158’, ‘Sm-159’, ‘Sm-160’, ‘Sm-161’, ‘Sm-162’, ‘Sm-163’, ‘Sm-164’, ‘Sm-165’, ‘Sn-115’, ‘Sn-116’, ‘Sn-117’, ‘Sn-117m’, ‘Sn-118’, ‘Sn-119’, ‘Sn-119m’, ‘Sn-120’, ‘Sn-121’, ‘Sn-121m’, ‘Sn-122’, ‘Sn-123’, ‘Sn-123m’, ‘Sn-124’, ‘Sn-125’, ‘Sn-125m’, ‘Sn-126’, ‘Sn-127’, ‘Sn-127m’, ‘Sn-128’, ‘Sn-128m’, ‘Sn-129’, ‘Sn-129m’, ‘Sn-130’, ‘Sn-130m’, ‘Sn-131’, ‘Sn-131m’, ‘Sn-132’, ‘Sn-133’, ‘Sn-134’, ‘Sn-135’, ‘Sn-136’, ‘Sn-137’, ‘Sr-100’, ‘Sr-101’, ‘Sr-102’, ‘Sr-103’, ‘Sr-104’, ‘Sr-85’, ‘Sr-85m’, ‘Sr-86’, ‘Sr-87’, ‘Sr-87m’, ‘Sr-88’, ‘Sr-89’, ‘Sr-90’, ‘Sr-91’, ‘Sr-92’, ‘Sr-93’, ‘Sr-94’, ‘Sr-95’, ‘Sr-96’, ‘Sr-97’, ‘Sr-98’, ‘Sr-99’,

T:

‘Ta-178’, ‘Ta-178m’, ‘Ta-179’, ‘Ta-179m’, ‘Ta-180’, ‘Ta-180m’, ‘Ta-181’, ‘Ta-182’, ‘Ta-182m’, ‘Ta-183’, ‘Ta-184’, ‘Ta-185’, ‘Ta-185m’, ‘Ta-186’, ‘Ta-187’, ‘Ta-188’, ‘Ta-189’, ‘Ta-190’, ‘Tb-156’, ‘Tb-156m’, ‘Tb-157’, ‘Tb-158’, ‘Tb-158m’, ‘Tb-159’, ‘Tb-160’, ‘Tb-161’, ‘Tb-162’, ‘Tb-163’, ‘Tb-164’, ‘Tb-165’, ‘Tb-166’, ‘Tb-167’, ‘Tb-168’, ‘Tb-169’, ‘Tb-170’, ‘Tb-171’, ‘Tc-100’, ‘Tc-101’, ‘Tc-102’, ‘Tc-102m’, ‘Tc-103’, ‘Tc-104’, ‘Tc-105’, ‘Tc-106’, ‘Tc-107’, ‘Tc-108’, ‘Tc-109’, ‘Tc-110’, ‘Tc-111’, ‘Tc-112’, ‘Tc-113’, ‘Tc-114’, ‘Tc-115’, ‘Tc-116’, ‘Tc-117’, ‘Tc-118’, ‘Tc-98’, ‘Tc-99’, ‘Tc-99m’, ‘Te-120’, ‘Te-121’, ‘Te-121m’, ‘Te-122’, ‘Te-123’, ‘Te-123m’, ‘Te-124’, ‘Te-125’, ‘Te-125m’, ‘Te-126’, ‘Te-127’, ‘Te-127m’, ‘Te-128’, ‘Te-129’, ‘Te-129m’, ‘Te-130’, ‘Te-131’, ‘Te-131m’, ‘Te-132’, ‘Te-133’, ‘Te-133m’, ‘Te-134’, ‘Te-135’, ‘Te-136’, ‘Te-137’, ‘Te-138’, ‘Te-139’, ‘Te-140’, ‘Te-141’, ‘Te-142’, ‘Th-227’, ‘Th-228’, ‘Th-229’, ‘Th-230’, ‘Th-232’, ‘Th-233’, ‘Th-234’, ‘Ti-50’, ‘Ti-51’, ‘Ti-52’, ‘Ti-53’, ‘Ti-54’, ‘Ti-55’, ‘Ti-56’, ‘Ti-57’, ‘Ti-58’, ‘Ti-59’, ‘Ti-60’, ‘Ti-61’, ‘Tm-166’, ‘Tm-167’, ‘Tm-168’, ‘Tm-169’, ‘Tm-170’, ‘Tm-171’, ‘Tm-172’, ‘Tm-173’, ‘Tm-174’, ‘Tm-175’, ‘Tm-176’, ‘Tm-177’, ‘Tm-178’, ‘Tm-179’,

U:

‘U-232’, ‘U-233’, ‘U-234’, ‘U-235’, ‘U-236’, ‘U-237’, ‘U-238’,

V:

‘V-50’, ‘V-51’, ‘V-52’, ‘V-53’, ‘V-54’, ‘V-55’, ‘V-56’, ‘V-57’, ‘V-58’, ‘V-59’, ‘V-60’, ‘V-61’, ‘V-62’, ‘V-63’, ‘V-64’, ‘V-65’,

W:

‘W-180’, ‘W-181’, ‘W-182’, ‘W-183’, ‘W-183m’, ‘W-184’, ‘W-185’, ‘W-185m’, ‘W-186’, ‘W-186m’, ‘W-187’, ‘W-188’, ‘W-189’, ‘W-190’, ‘W-190m’, ‘W-191’, ‘W-192’,

X:

‘Xe-128’, ‘Xe-129’, ‘Xe-129m’, ‘Xe-130’, ‘Xe-131’, ‘Xe-131m’, ‘Xe-132’, ‘Xe-132m’, ‘Xe-133’, ‘Xe-133m’, ‘Xe-134’, ‘Xe-134m’, ‘Xe-135’, ‘Xe-135m’, ‘Xe-136’, ‘Xe-137’, ‘Xe-138’, ‘Xe-139’, ‘Xe-140’, ‘Xe-141’, ‘Xe-142’, ‘Xe-143’, ‘Xe-144’, ‘Xe-145’, ‘Xe-146’, ‘Xe-147’,

Y:

‘Y-100’, ‘Y-100m’, ‘Y-101’, ‘Y-102’, ‘Y-102m’, ‘Y-103’, ‘Y-104’, ‘Y-105’, ‘Y-106’, ‘Y-107’, ‘Y-88’, ‘Y-88m’, ‘Y-89’, ‘Y-89m’, ‘Y-90’, ‘Y-90m’, ‘Y-91’, ‘Y-91m’, ‘Y-92’, ‘Y-93’, ‘Y-93m’, ‘Y-94’, ‘Y-95’, ‘Y-96’, ‘Y-96m’, ‘Y-97’, ‘Y-97m’, ‘Y-98’, ‘Y-98m’, ‘Y-99’, ‘Yb-169’, ‘Yb-170’, ‘Yb-171’, ‘Yb-171m’, ‘Yb-172’, ‘Yb-173’, ‘Yb-174’, ‘Yb-175’, ‘Yb-175m’, ‘Yb-176’, ‘Yb-176m’, ‘Yb-177’, ‘Yb-177m’, ‘Yb-178’, ‘Yb-179’, ‘Yb-180’, ‘Yb-181’,

Z:

‘Zn-66’, ‘Zn-67’, ‘Zn-68’, ‘Zn-69’, ‘Zn-69m’, ‘Zn-70’, ‘Zn-71’, ‘Zn-71m’, ‘Zn-72’, ‘Zn-73’, ‘Zn-73m’, ‘Zn-74’, ‘Zn-75’, ‘Zn-76’, ‘Zn-77’, ‘Zn-77m’, ‘Zn-78’, ‘Zn-79’, ‘Zn-80’, ‘Zn-81’, ‘Zn-82’, ‘Zn-83’, ‘Zr-100’, ‘Zr-101’, ‘Zr-102’, ‘Zr-103’, ‘Zr-104’, ‘Zr-105’, ‘Zr-106’, ‘Zr-107’, ‘Zr-108’, ‘Zr-109’, ‘Zr-91’, ‘Zr-92’, ‘Zr-93’, ‘Zr-94’, ‘Zr-95’, ‘Zr-96’, ‘Zr-97’, ‘Zr-98’, ‘Zr-99’.

Each of the nuclides and their associated contributions to decay heat, gamma emissions and spontaneous fission rates are reported at various combinations of fuel BIC. It is worth noting that while signatures such as DH, SF, and GS were included in the dataset as individual contributions from all nuclides, the (ɑ, n) neutron emission rate has been included as a sum over all nuclides. This is because (ɑ, n) reactions require a source nuclide (for the ɑ-decay) and a target nuclide (which undergoes (ɑ, n) reactions) and inclusion of every source-target combination and their resulting (ɑ, n) emissions would be impractical and would greatly increase the size of the resulting dataset. The BU values were chosen in a manner that there was a total of 75 BU steps starting at 10^−9^ MWd/kgU (as it corresponds to 1 second of fuel irradiation) and going up to roughly 29 MWd/kgU. Similarly, the IE parameter varies between 10.0 and 20.0 wt. % U-235 in steps of 0.25 which implies that there are 41 steps covering the full range of IE in total. The motivation behind choice of step size 0.25 for IE is attributed to the choice of step sizes in [2] and also allows for a grid size that is fine enough to accommodate possible deviations in the selected IE value for the chosen MSR concept in the future. Lastly, for CT, the nuclide inventories and their associated contributions to DH, SF, and GS were computed at a total of 101 steps. These CT values range between roughly 90 days and up to 40 years. Since the chosen MSR concept has not been deployed yet, the choice of CT step size was based on the need for having a CT grid that would be fine and broad enough to give adequate information of salt properties at various CT values. These parameters are summarized in Table 2.

**Table 2:** Key dataset parameters used for setting up Serpent cases.

Parameter Name	Parameter Value
Burnup (B)	Range: 0.0 – 29.0 MWd/kgU 75 steps in total For BU < 1.0 MWd/kgU – steps increasing by a factor of 2.5 For BU 1.0 MWd/kgU – steps of 0.5 MWd/kgU
Initial enrichment (I)	Range: 10.0 – 20.0 wt. % U-235 41 steps in total Steps of 0.25 wt. % U-235
Cooling time (C)	Range: 0 – 40 years 101 steps in total For CT < 10 y – steps of 0.25 y For CT ≥ 10 y – steps of 0.5 y

The dataset can be read in using the popular python data processing library named Pandas with read_csv with the ‘pyarrow’ engine which makes this step faster. One may further use the ‘usecols’ option to selectively read in the required columns of data to reduce memory usage and time required for reading in the dataset. Once the data has been read in, one can create smaller subsets of the data:

import pandas as pd

df = pd.read_csv(‘path/to/dataset.csv’, engine=‘pyarrow’, usecols=[‘BU’,‘CT’,‘IE’,‘U235’,‘Pu239’,‘SF’,‘AN’])

The user may then create subsets of the dataset by selecting the enrichment and cooling time (IE = 15.0 wt. % U-235 and CT = 0.0 in this case) as follows:

dfIE15CT0 = df[(df[‘IE’]==15.0) & (df[‘CT’]==0.0)]

### 3.1 Experimental Design, Materials and Methods

The burnup calculations carried out for creation of the dataset consists of a 3D model of a molten salt reactor core that represents the CMSR design [6] proposed by Seaborg. The reactor is an advanced nuclear reactor characterized by the use of a liquid fluoride molten salt fuel and a liquid sodium hydroxide moderator. The CMSR consists of multiple subsystems that will be integrated on a reactor barge or vessel, which is expected to be towed to and anchored at the site of the reactor barge and connected to the grid on-site. One CMSR unit is proposed to generate up to 250 MWth of superheated steam for producing electrical power to the onshore electrical grid and the barge is expected to house up to four such CMSR units. It is expected that the reactor fuel cycle will be nearly 12 years long and the fuel will reach a terminal burnup of roughly 20 MWd/kgU at End of Life (EOL). The reactor is designed to operate as a thermal spectrum converter fueled with NaF-KF-UF_4_ fuel and sodium hydroxide as the moderator. The reactor core internals are designed with a proprietary nickel-based alloy which is somewhat similar to INOR alloy, or Hastelloy-N used in past experimental molten salt reactors such as the Molten Salt Reactor Experiment (MSRE) [7]. This material was designed with the aim to withstand the highly corrosive nature of the salt and the moderator. A proprietary protective coating composed of a high nickel content alloy is included which greatly reduces the rates of corrosion and further extends the lifetime of the reactor components. The salt is expected to circulate in the core in 235 fuel salt channels each of which is surrounded by the moderator. The full range of reactor core parameters including those that were used in the Serpent model supplied by Seaborg Technologies are shown in Table 3.

**Table 3:** Model parameters (from [6]) for the CMSR core including those used in Serpent calculations

Parameter Name	Parameter Value
Thermal power rating	250 MW
Reactor type	Molten Fluoride Salt
Operating temperature	650℃
Neutron spectrum	Thermal
Moderator	Sodium Hydroxide (NaOH)
Salt type	FUNaK
Primary driver	Uranium-235
Structural material	Proprietary nickel-based alloy
Salt mass (in reactor)	105t (approx.)
Salt volume (in reactor)	25 m^3^
Salt density	4200 kg/m^3^
Enrichment	Ideally < 12 %. (Range used 10–20 %)
Time in reactor	12 years
Maximum burnup	18 MWd/kgU approx. (Range used 0–29 MWd/kgU)
Online refueling	No
Off-Gas System (OGS)	Yes (not used in Serpent model)

The dimensions of the critical components in the CMSR core such as the fuel salt channel, the cladding and the overall core are given in Table 4.

**Table 4:** Model dimensions for the CMSR core components used in the Serpent calculations.

Parameter Name	Parameter Value
Number of salt channels	235
Channel radius	5 cm
Cladding inner radius	5.1 cm
Cladding outer radius	5.5 cm
Coating thickness	0.1 cm
Fuel tube radius	5.4 cm
Overall core geometry	Hexagonal

**Fig. 1:**
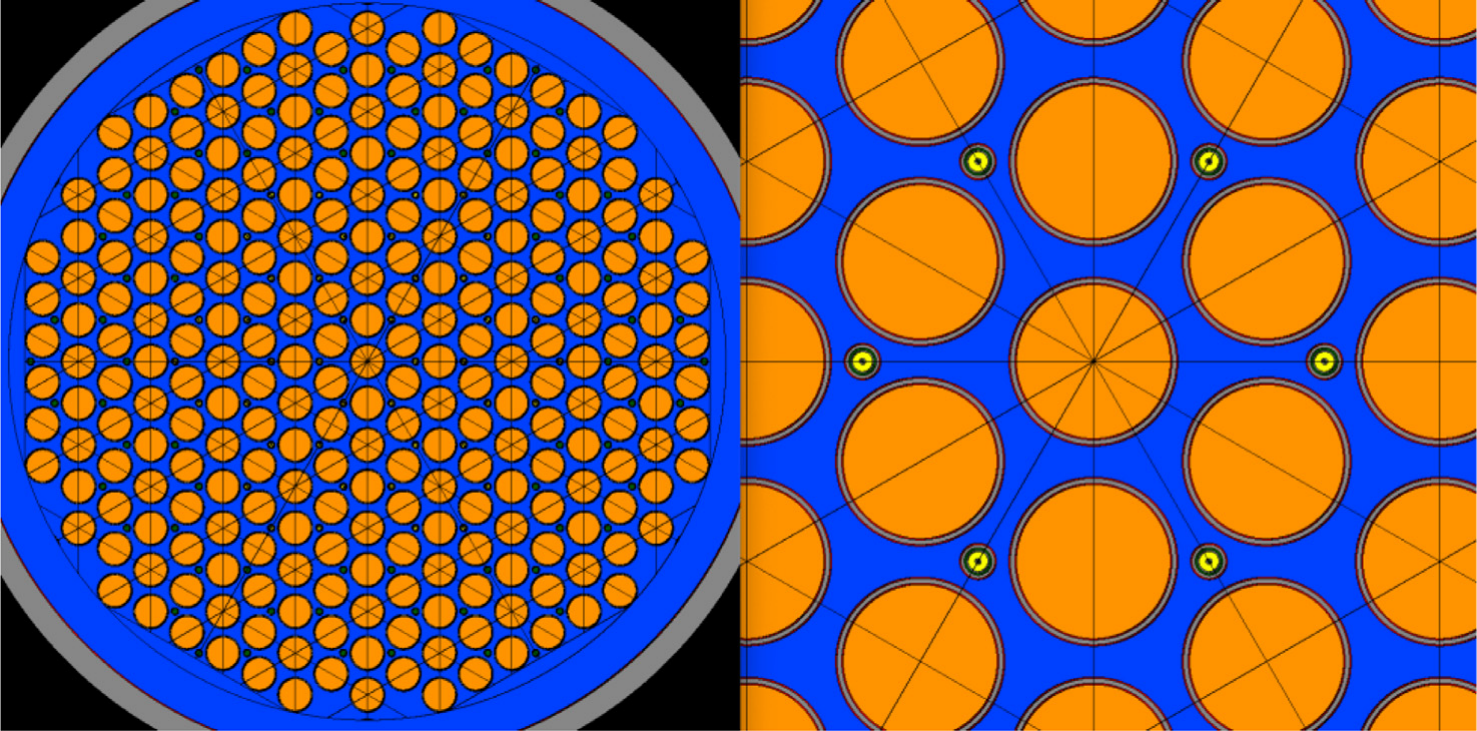
Left: Radial cross-section of the Serpent model for the CMSR core showing the hexagonal arrangement of fuel salt channels (orange), structural material in gray, helium in light green, the control rod locations (smaller circles between fuel channels), and moderator (deep blue). Right: Closer view of radial cross-sections of the fuel salt channel and control rod tubes. (For interpretation of the references to color in this figure legend, the reader is referred to the web version of this article.)

While the CMSR is also expected to have an Off-Gas System (OGS) which is capable of removing highly corrosive fission products and other fission poisons^1^, for simplicity, the OGS has not been included in the Serpent model of the CMSR core. Moreover, material flows of any nature (be it addition of fissile material mid-cycle or removal of volatile and non-volatile FPs) are not modeled in Serpent for the creation of this database. Non-inclusion of fission product removal from the primary salt is likely to have an adverse impact on the fuel cycle by affecting the neutron economy as some of the fission products have high neutron absorption cross-sections. As far as reactivity control in the core is concerned, it is achieved through a chemical shim^2^ (boron) which is directly dissolved in the moderator as well as control rod banks that are suitably located in different regions of the core. The core consists of three different control rod regions, all of which employ boron carbide rods to control reactivity. The three different types of rods correspond to emergency shutdown banks and for compensating depletion of fissile material with burnup. The control rods are included as such in the Serpent model of the core as 100 % B-10 carbide. The control rods have not been moved during depletion and this may introduce a bias in the overall neutron spectra during the cycle. The core design and arrangement of fuel salt channels and control rod guide tubes are shown in Fig. 1.

For the Monte–Carlo calculations in Serpent, the JEFF3.3 [7] neutron interaction data, radioactive decay data, fission yields data have been used in the Serpent calculations. Owing to lack of thermal scattering law (TSL) data for NaOH, the moderator has been modeled as a free gas. Since the model used in the Serpent burnup calculations represents a 3D model of the full core, vacuum boundary conditions are used.

**Fig. 2:**
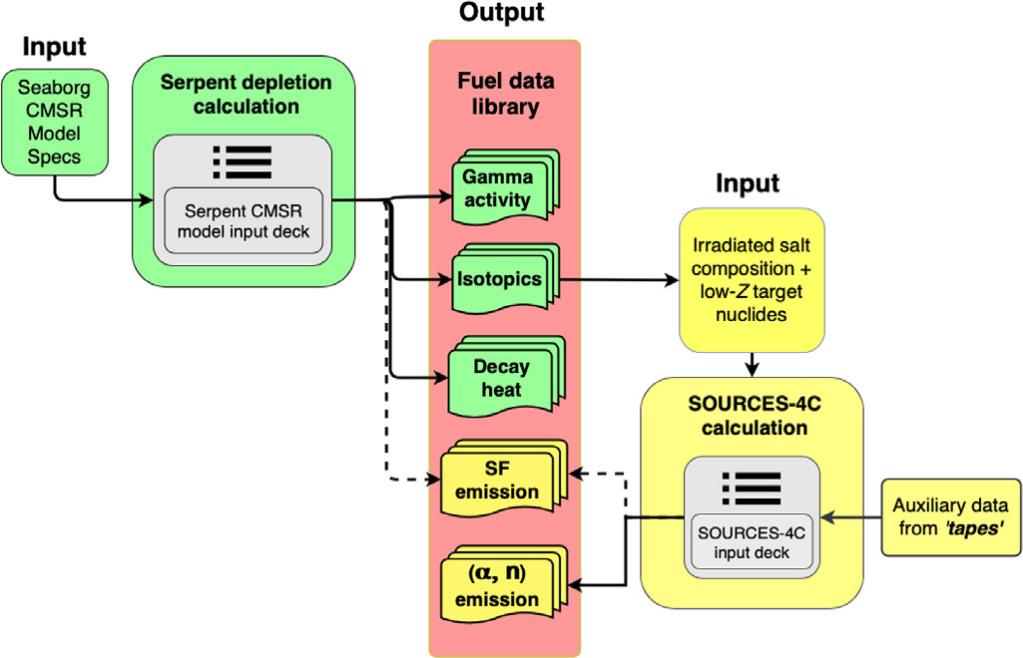
Flow chart showing calculations carried out in Serpent2 and SOURCES 4C for setting up the fuel library.

For calculations carried out in the code SOURCES 4C, the output files generated using Serpent calculations were used as input to compute the neutron emission rates. SOURCES 4C allows user to create input definitions with atomic fractions of ɑ-emitting nuclides and isotopic stoichiometric fractions for low-Z nuclides that have non-zero cross-section values for (ɑ, n) reactions. Thereafter, it uses supplementary data supplied in the software package as *`tapes’* in order to compute neutron emission rates from spontaneous fissions and from (ɑ, n) reaction channels. The details of the methodology have been shown in Fig. 2.

## Limitations

The dataset of irradiated molten fuel salt composition at various stages of irradiation and cooling time has been presented herein. The dataset is expected to be representative of fuel salt irradiation in MSRs of similar design, features (such as no online refueling or removal of gaseous and volatile fission products), and operation. Users of this dataset must employ due deliberation and are advised to exercise caution while using this dataset for reactors that have designs that deviate significantly from the one used in preparation of the dataset. Users are also cautioned against loading the entire dataset at once if there are memory concerns and use the author's suggestions to load in only the required columns of the dataset.

## Ethics Statement

(1) This material is the authors' own original work, which has not been previously published elsewhere. 2) The paper is not currently being considered for publication elsewhere. 3) The paper reflects the authors' own research and analysis in a truthful and complete manner. 4) The paper did not involve any human or animal studies and did not involve collection of social media data.

## CRediT authorship contribution statement

**Vaibhav Mishra:** Conceptualization, Methodology, Software, Writing – original draft, Investigation. **Zsolt Elter:** Conceptualization, Methodology, Software, Resources, Writing – original draft, Supervision. **Erik Branger:** Conceptualization, Methodology, Writing – original draft, Supervision. **Sophie Grape:** Conceptualization, Methodology, Writing – original draft, Supervision, Funding acquisition, Project administration. **Sorouche Mirmiran:** Conceptualization, Writing – review & editing.

## Data Availability

Irradiated fuel salt data library for a molten salt reactor produced with Serpent2 and SOURCES 4C codes (Original data) (Mendeley Data).

## Footnotes

1 Thereby maintaining the fuel salt chemistry and improving the overall neutron economy in the core

2 Serpent model does not include the chemical shim in the moderator and reactivity control is modeled by presence of control rod material.

## References

[1] Rossa R. (2019).

[2] Elter Zs. (2020).

[3] Leppänen J. (2013). *“Serpent–a continuous-energy Monte Carlo reactor physics burnup calculation code”*. VTT Tech. Res. Centre Finland.

[4] Wilson W.B., Perry R.T., Charlton W.S., Parish T.A. (2009). “*Sources: A code for calculating (alpha,* n)*, spontaneous fission, and delayed neutron sources and spectra”*. Progress Nucl. Energy.

[5] Rearden B.T., Jessee M.A. (2018).

[6] Pater M., Stampe J., Pettersen E.E. (2022). WCFS2020.

[7] Plompen A.J.M., Cabellos O., De Saint Jean C. (2020). *“The joint evaluated fission and fusion nuclear data library“*, JEFF-3.3’. Eur. Phys. J. A.

